# 

**Published:** 1888-01

**Authors:** 


					﻿Entered at the Post Office, at Austin, Texas, as Second Class Mail Matter
Vol. III
JANUARY, 1888
No. 7.
DANIEL'S
MEDICAL
JOURNAL
F. E. DANIEL., M. D., Editor and Publisher, AUSTIN, TEXAS.
Northern office, 1217 Filbert Street, Philadelphia.
Steam Book and Jor Printer, Austin, Texas
				

